# The Week's Work

**Published:** 1911-05-20

**Authors:** 


### The Week's Work.

### THE BRITISH HOSPITALS ASSOCIATION AND THE CRISIS.

Whatever opinions may be held upon the subject of Mr. Thies' general forecast as to the effects of the Insurance Bill on hospitals, which he gives in a special interview this week—and no one will deny their great interest and authority—his concluding suggestion for a general conference upon the subject is certain of a welcome. Only by active discussion can hospital authorities throughout the country comprehend with any degree of certainty the effects of this intricate measure, and without such discussion we may be landed in a position from which it would be too late to retreat. That such discussion may not be wasted, and may represent authoritatively the front rank of hospital opinion, it is all-important that it should be conducted under proper auspices, and by a body which will command for its conclusions general acquiescence and respect. It seems to us, therefore, that the British Hospitals Association, which, as already announced, meets at Manchester on September 28 and 29, should take the lead in this matter, and, if necessary, summon a special session to discuss the Insurance Bill in all its aspects from the institutional point of view. What the hospitals want at the moment is a lead. No other body is so fitted as the British Hospitals Association to give it. With the Association largely rests the great responsibility of the present moment that the hospitals' best interests may be safeguarded amid the new condition that the Insurance Bill, even if much modified, is certain to create. We hope that Dr. Mackintosh and Mr. West, its honorary secretaries, will act as energetically as the situation demands, and that no time may be lost before the British Hospitals Association makes known its arrangements for discussion and direction on this matter.

### MR. A. W. WEST ON THE WARD UNIT.

Although to St. George's men the name of Mr. A. W. West has been familiar for many years, to the public at large he first became prominent some eight or nine years ago as the collaborator with Dr. A. C. Latham in the successful designs for the £500 prize offered for open competition in connection with the late King's Sanatorium for Consumption, since then constructed and opened at Midhurst. It will be remembered that by the conditions of the competition each entry was to be the joint work of a physician and an architect; and nothing was more natural than that Dr. Latham should avail himself of the combined architectural and administrative experience possessed by Mr. West, who had been for some time one of the most active and useful members of the Governing Board, and was also a practising architect. More recently Mr. West has been before the public as the apologist—using the word in the Socratic sense—of the Hospital in the dispute as to the exact allocation of certain funds between the Hospital and its medical school. As Joint Treasurer, at the weekly Board, and by the constant personal interest which he has always taken in the administrative details of the work of the Hospital, Mr. West has established a claim upon the respect of those who love his Hospital far greater and more enduring than most people outside its circle are aware of. In another column (p. 197) appears a contribution by Mr. West to the series of articles on the Ward Unit, which has evoked so much interest and discussion lately among hospital experts.

### HOSPITAL HISTORIES AND OUR READERS.

The first volume of the History of St. Bartholomew's Hospital is now, we hear, in print, and the complete work is expected to be published between now and December. Some idea of the scale on which this is being carried out may be gained from the fact that the first volume alone will contain more than six hundred pages. In this connection we take the opportunity of thanking those hospital secretaries who have complied with the request which we published on April 22, page 91, and have sent the title, price, and publisher's name of any history of their institution which has been written. In case our previous notice has escaped the attention of any, let us repeat that "whether the history has been written in two large volumes or summarised in an authoritative monograph, information concerning it will be equally welcome." This is wanted so that a complete bibliography may be available here for general information, and we especially thank those who have gone out of their way to record particulars of hospital histories other than their own.

### THE ST. BARTHOLOMEW'S HOSPITAL ESTATES.

The Finance Act of 1910, according to the Treasurer's report, has hit St. Bartholomew's Hospital pretty severely. Owing to its possession of land, both reversion duty and undeveloped land duty is liable to be paid by the hospital whenever such land cannot be held to be actually occupied for hospital purposes. St. Bartholomew's also derives an appreciable portion of its invested income, or endowment, from landed property, and increment duty will shortly be demanded of it on the grant of any lease for a longer period than fourteen years. Now it so happens that St. Bartholomew's is one of those hospitals which have been counting on an improved rent-roll, when certain leases fell due, to help towards paying its capital debt. This increment value, however, as the result of last year's Finance Act, will have to pay its toll to the State. Though the country may be held to have approved this taxation of rent, we cannot escape the moral that it does not do for a hospital to be a landlord nowadays. Direct taxation is increasing; and a glance at the opinions expressed by hospital secretaries on another page as to the effects of the new Insurance Bill makes it clear that the fear of direct subsidy may be no less dangerous.

### RESEARCH AT A MENTAL HOSPITAL.

A remarkable amount of feeling has been aroused locally against the municipal authorities of the Cardiff Mental Hospital for proposing to sanction a considerable expenditure upon a research department in connection with the institution. The protest has been of two kinds, one being from ratepayers who object to further expenditure in general, the other being from citizens, whether ratepayers or not, who object to expenditure upon research. It seems that additional feeling has been stirred on the ground that the new departure was sanctioned before any except those immediately interested in it had had an opportunity of expressing their opinion on the matter. It is at least clear that research, which is not very popular, under municipal auspices has very little chance of surviving. In view, however, of the conflicting opinion as to its efficacy in assisting the study of mental pathology, the hospital's friends can hardly refrain from saying that it has not managed its affairs very tactfully at the present juncture. For one reason or another the Cardiff Mental Hospital has been the centre of so much disputation lately, that it is consoling to remember that it does not depend for its daily maintenance upon the voluntary subscriptions of the public.

### A BELATED "MEMORIAL" SCHEME.

The fourth annual meeting of the Jewish Hospital Association (Limited) this week reveals the fact that the scheme for a hospital for Jews in London is still being laboriously proceeded with. Fortunately, it is receiving very little support, the public being very well aware that the excellent Jewish wards at the London, and the extensive patronage given by Jews to the Metropolitan and German Hospitals, more than supply the needs of Jewish patients. Indeed, it is safe to say no other class of patient is so well catered for. We dealt with the whole question in The Hospital of October 1, 1910, page 30; and if reasoned argument could have convinced the scheme's promoters of its want of usefulness, and of the difficulties it is likely to create for the very institutions which have done so much for Jewish patients, it would have been dropped. It was not dropped, because the site had been ordered at that time, though not paid for, and the present impasse is such that its promoters would find almost equal difficulty in abandoning as in proceeding with the scheme. The irony of the situation lies in the scheme ever having been advocated as a memorial to King Edward, to the very man, in other words, who by his own Fund created every bond he could to unite the hospitals, and to centralise them, and who, we are bold to say, would have immediately recognised the fact that if he gave his patronage to a separatist scheme for a Jewish hospital, movements would be started for Catholic, Nonconformist, Moslem, Anglican, and even Vegetarian hospitals. The scheme will not bear examination, as indeed seems to be felt by its promoters, since they talk about the importance of their scheme, and with the next breath confess that it is only for fifty beds. If the Jews took it seriously, ten times that number would be necessary, but to claim importance to the Jewish Colony in London for a hospital of such meagre size is enough in the eyes of experienced men to make the scheme ridiculous. We have done our best to help Dr. Gaster and Mr. Berliner out of their fix by suggesting, in the issue mentioned, the statesmanlike way of disposing of the funds subscribed.

### HOSPITAL OFFICERS VISIT TO LONDON.

The recent quiet visit of the Manchester Branch of the Hospital Officers' Association to London achieved several very useful results. It really did produce as much comparison of method as is possible within the limits of a day's travel. The system for the registration of out-patients at the Great Ormond Street Hospital and at the London were explained by Mr. Stewart Johnson and by Mr. Morris, and an opportunity of personal intercourse was given which may lead to closer inquiries and more ready answers than might result from simple letter writing between Manchester and London. In spite, too, of the hospitality of Mr. Alvey's luncheon and of Lord Sandhurst's tea, which, as treasurer, he provided at St. Bartholomew's, the visit did not degenerate into a social function. By bringing together such superintendents as Mr. Carnt, of Manchester, Mr. Arthur Griffiths, of Ipswich, Mr. Ruddle, of Salford, Mr. Blake, of Oldham, Mr. Shaw, of Southport, Mr. Smith, of Blackburn, Major Tyler, of Stockport Infirmary, and Mr. Ratcliffe, of St. Mary's, Manchester, with those of our London Hospitals, a uniformity, not merely of accounts, is made possible and easy to strengthen. The value of such interchange of experience was recognised, of course, when the Hospital Officers' Association was founded. Such visits as this last find their value in making that interchange a matter of friendly habit.

### THIS WEEK'S DRUG MARKET.

A fair amount of business has been done in the drug market during the week, and there are signs that a further improvement may set in shortly. The hot weather has caused an increase in the demand for such articles as cream of tartar and citric and tartaric acids, with the result that prices show some inclination to advance, although up to the present there is no change in the quotations. The price of quicksilver is unaltered but a reduction is expected, and when this takes place it may be followed by a reduction in the prices of calomel and other sorts of mercury. English refiners of camphor have reduced their prices by 1d. per pound. Cod-liver oil is still declining in value, and the demand is extremely quiet. An advance in the price of balsam tolu is expected, and balsam copaiba also shows a tendency to advance. Business has been done in buchu leaves at dearer rates. Ergot has again advanced in price, and high prices are asked for jalap. Croton oil and dill oil are dearer. The price of opium is very firmly maintained, and makers of codeine now quote higher prices.

